# Altitudinal Gradient of Microbial Biomass Phosphorus and Its Relationship with Microbial Biomass Carbon, Nitrogen, and Rhizosphere Soil Phosphorus on the Eastern Slope of Gongga Mountain, SW China

**DOI:** 10.1371/journal.pone.0072952

**Published:** 2013-09-05

**Authors:** Hongyang Sun, Yanhong Wu, Dong Yu, Jun Zhou

**Affiliations:** 1 Key Laboratory of Mountain Surface Processes and Ecological Regulation, Chengdu Institute of Mountain Hazards and Environment, Chinese Academy of Sciences, Chengdu, China; 2 University of Chinese Academy of Sciences, Chinese Academy of Sciences, Beijing, China; University of Illinois, United States of America

## Abstract

Microbial biomass phosphorus (MBP) is one of the most active forms of phosphorus (P) in soils. MBP plays an important role in the biogeochemical P cycle. To explore MBP distribution and its relationship with other factors, the MBP and rhizosphere soil P concentrations and fractions in six vegetation zones on the eastern slope of Gongga Mountain in SW China were investigated. The MBP distribution followed a parabolic pattern with altitude and the concentration was highest in the subalpine dark coniferous forest (SDC) zone, which was approximately 3500 m above sea level (asl). Below 3500 m asl, the MBP distribution was controlled by precipitation and vegetation type. In addition, temperature, precipitation and vegetation type controlled the MBP distribution at elevations above 3500 m asl. No specific distribution pattern was determined regarding rhizosphere soil P fractions. However, MBP was significantly correlated with the unavailable P fraction in the rhizosphere rather than with the available P fraction. This result suggests that the relationships between the rhizosphere soil P fractions and the MBP depend on time. The microbial biomass element ratios were relatively consistent on the eastern slope of Gongga Mountain. However, variations in the microbial biomass element rations were observed in six of the vegetation zones. The mean C:N:P ratio was 9.0∶1.3∶1. Overall, vegetation type resulted in the observed fluctuations of the microbial biomass element ratio.

## Introduction

Phosphorus (P) is an essential element that controls plant growth and primary production in terrestrial ecosystems [1]. Therefore, the bioavailability of P in terrestrial ecosystems is important and should be understood. Previous research proved that P in natural ecosystems was mainly sourced from rock weathering, and transformed during biogeochemical processes before being available for ecosystems [2], [3]. Microbial activity is an important component of this biogeochemical process. Thus, soil microbial biomass phosphorus (MBP) provides bioavailable P to terrestrial ecosystems [4]. However, the turnover of MBP is largely due to the short life span of microorganisms. Therefore, MBP is one of the most active components in soils [4]–[6] and is an important source of bioavailable P in ecosystems [4], [7]. According to Brookes et al. [8], the turnover rate of MBP in England is approximately 2.5 yrs, and the turnover rate in Chinese red soils is much faster (0.36–0.59 yrs) [9]. Moreover, some of the bioavailable P may be retained by microbes, which will affect P chelation and exchange reactions [10], [11].

Soil pH, temperature, moisture and vegetation type are important factors that influence microbial activity [12], [13]. Lindo and Visser [14] concluded that microbial biomass was significantly different in deciduous forests relative to clear-cut and uncut control forests. A controlled-climate experiment confirmed the effects of temperature on microbes. For example, microbial N, P and S increased during the winter and through the early spring before decreasing [15]. Regarding microbial activity, soil moisture is just as important as soil temperature. Soil microbial biomass is greater in soils with greater moisture contents [16]. Microbial variation is significant during rapid changes in soil moisture conditions (e.g., drying and rewetting cycles) [17]. However, in some ecosystems, P and/or bioavailable P concentrations may limit microbial processes in soils [18].

Environmental factors should be considered when studying microbial geochemical P cycles. Most studies that have considered microbial geochemical P cycles were conducted under controlled environmental conditions. However, a few of these studies were designed in natural ecosystems (e.g., mountain systems). Gongga Mountain (29°20′-30°20′ N, 101°30′-102°15′ E) is an ideal area for investigating the relationships between microbes and the biogeochemical P cycle due to its altitude, integrated vegetation succession and temperature and precipitation gradients. Here, the relationships between microbial biomass and rhizosphere soil P with other environment factors are discussed by investigating the MBP and rhizosphere soil P gradients with altitude on the eastern slope of Gongga Mountain. This analysis was conducted to explore the interaction between the microbes and the P cycle in the mountain ecosystem.

## Materials and Methods

### Ethics Statement

All necessary permits were obtained for the described field studies. We conducted this study at the Alpine Ecosystem Observation and Experiment Station on Gongga Mountain, which belongs to the Institute of Mountain Hazards and Environment at the Chinese Academy of Sciences & Ministry of Water Conservancy. We obtained permission from the station and institute to use the sample plots. Furthermore, our study did not harm the environment and did not involve endangered or protected species.

### Study Site

**Figure 1:**
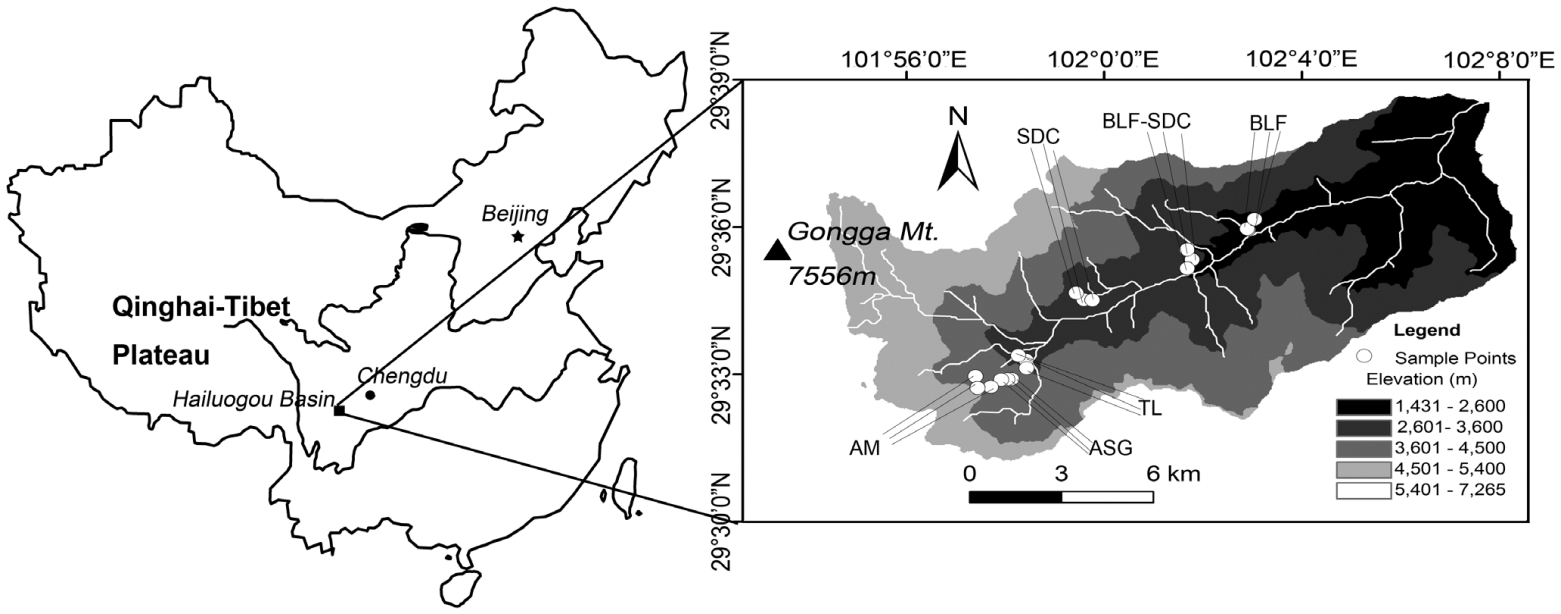
Location of the sampling sites on the eastern slope of Gongga Mountain, SW China. BLF: broad-leaf forest; BLF-SDC: broad-leaf and dark coniferous mixed forest; SDC: subalpine dark coniferous forest; TL: timberline (forest coverage<10%); ASG: alpine shrub-grass; and AM: alpine meadows.

Gongga Mountain is located on the southeastern edge of the Tibetan Plateau and has a summit elevation of 7556 m asl (Fig. 1) [19]. Gongga Mountain has a temperate monsoon climate with a monthly mean temperature of between-4.5°C in January and 12.7°C in July. The mean annual precipitation is 1949 mm at 3000 m asl with a mean annual potential evaporation of 264 mm. Precipitation increases with increasing elevation up to 3500 m asl, but decreases between 3500 and 4200 m asl. Above 4000 m asl, the main form of precipitation is snow. The snowline appears at approximately 4900 m asl The lowest elevation on the eastern slope of Gongga Mountain occurs in the Dadu River valley (approximately 1300 m asl). The climate in this region is subtropical arid and hot.

Natural forests mainly occur between 2000 and 3600 m asl on Gongga Mountain. Between 2000 and 2400 m asl, the broad-leaf forest (BLF) is dominated by *Lithocapus cleistocarpus, Acer flabellatum* and other species. *Abies fabri* and *Acer flabellatum* are the dominant trees in the broad-leaf and dark coniferous mixed forests (BLF-SDC, from 2400 to 2900 m asl). *Abies fabri* is the dominant tree species in the subalpine dark coniferous forests (SDC, from 2900 to 3650 m asl). The timberline (TL) appears at approximately 3700 m asl and includes *Abies fabri*, *Rhododendron* and other species. The alpine shrub-grass zone (ASG) extends from 3750 to 4100 m asl. In addition, the alpine meadow zone (AM) extends from 4100 m asl to the snowline. These zones and their soil properties are listed in Table 1.

**Table 1:** Soil and vegetation properties in the six vegetation zones.

Vegetation zone	BLF	BLF-SDC	SDC	TL	ASG	AM
Elevation(m asl)	2361	2777	3317	3715	4015	4221
Dominant	*Lithocapus*	*Abies fabri;*	*Abies fabri*	*Abies fabri*	*Rhododendron sp.*	*Kobresia pygmaea;*
plant species	*cleistocarpus;*	*Acer flabellatum*		*Rhododendron sp.*		*Allium prattii*
	*Acer flabellatum*					
Soil	Brown soil	Brown soil	Dark brown soil	Subalpine bleach	Alpine	Alpine meadow
				spodosol	shrub-meadow soil	soil
pH	5.50±0.41	4.49±0.18	3.44±0.02	3.99±0.12	4.92±0.42	4.94±0.17
aSOM(mg g^−1^)	70.65±9.05	55.38±2.32	59.47±7.09	50.41±7.60	28.75±3.45	76.40±16.50
Soil moisture(%)	235.8±22.4	339.6±37.1	264.9±33.3	229.2±21.2	274.4±49.8	85.7±14.0
bSoil temp(°C)	14.1±0.3	11.0±0.1	9.5±0.1	8.6±0.4	8.5±0.4	7.7±0.1
Total N(mg g^−1^)	2.34±0.17	1.73±0.05	2.01±0.12	1.88±0.13	1.02±0.08	1.11±0.28
Total P(µg g^−1^)	462.29±30.46	637.86±32.56	511.52±51.01	449.97±24.11	498.70±168.3	640.00±90.31
Organic P(µg g^−1^)	160.79±18.16	344.46±55.38	250.05±24.82	238.68±8.37	332.17±68.50	360.92±63.13
Al(mg g^−1^)	22.00±6.93	30.66±9.13	32.72±4.33	31.69±10.77	57.29±1.95	55.00±6.30
Ca(mg g^−1^)	26.79±2.96	9.67±0.78	12.26±1.74	13.36±2.53	27.32±0.96	25.67±1.85
Fe(mg g^−1^)	10.59±1.84	15.81±4.26	18.73±3.62	15.50±3.51	38.90±2.02	37.08±4.32
K(mg g^−1^)	5.31±0.94	10.01±3.49	9.40±0.82	10.39±4.01	19.74±0.49	18.29±2.21

a SOM: soil organic matter;

b Soil temp: soil temperature.Values are means ± SE (n = 3). BLF: broad-leaf forest; BLF-SDC: broad-leaf and dark coniferous mixed forest; SDC: subalpine dark coniferous forest; TL: timberline (forest coverage<10%); ASG: alpine shrub-grass; and AM: alpine meadows.

### Sampling

In September 2010, soil samples were collected separately from the six vegetation zones on the eastern slope of Gongga Mountain (Fig. 1). Three plots were selected based on the “Protocols for Standard Soil Observation and Measurement in Terrestrial Ecosystems” [20]. Briefly, intact areas in each vegetation zone with flat terrain, zonal vegetation and soils were selected. In each of these zones, three plots (2 m×2 m,>20 m intervals among the plots) were established. To collect the rhizosphere soils from the six vegetation zones, typical and zonal vegetation were selected (separately) and the roots were dug out. For example, *Abies fabri* is typical in subalpine dark coniferous forests. Thus, fine rootlets in mature *Abies fabri* forests (diameter 0.1–0.5 cm, root density >0.6 cm cm^−3^) were tracked and extracted at a depth of 0–30 cm within these plots. The soil that adhered to the roots was collected, placed in polyethylene plastic bags and homogenized to obtain a representative sample for each plot. Samples were stored at 4°C prior to analysis.

### Chemical and Biochemical Measurements

After the large rocks and plant remains were removed, the moist soil samples were passed through a 2 mm sieve and separated into two portions. One portion was used to measure soil chemical properties (e.g., pH and soil P) and the other portion was used for the MBP, microbial biomass C (MBC) and microbial biomass N (MBN) measurements.

Sequential chemical extractions for P in the rhizosphere soil was conducted according to the methods described by Hedley et al. [21] and Agbenin et al. [22]. First, 0.5 g of air-dried soil was passed through a 0.15 mm sieve before weighing and placing into a centrifuge tube. After shaking the tube for 16 h with 30 ml of deionized water and two resin strips (area: 1 cm×3.5 cm, Anion 204 UZRA), the resin-Pi was extracted and measured. Next, 0.5 M NaHCO_3_ was added to the tube to extract inorganic and organic P (NaHCO_3_-Pi and NaHCO_3_-Po, respectively). NaHCO_3_-Pi, NaHCO_3_-Po and resin-Pi are the most bioavailable P fractions [23]. Thereafter, 0.1 M NaOH was added to the tube to extract inorganic and organic P (NaOH-Pi and NaOH-Po, respectively). These P are less labile and are adsorbed onto Fe or Al oxides and humic compounds in soils [24]. Finally, the concentrated HCl was added to the remaining soil to extract HCl-Pi and HCl-Po, which included P in apatite and other non-labile forms. The organic P digestion was based on the persulfate digestion method [25] and the inorganic P concentrations were determined with the ascorbic acid molybdenum blue method [26].

MBP was measured with the fumigation extraction method [27]. First, 10.0 g of the moist soil sample was fumigated with ethanol-free chloroform. After chloroform removal, the P in the soil sample was extracted with sodium bicarbonate (200 ml, pH 8.5) for 30 min while shaking at 300 rpm. The suspension was centrifuged (8000 rpm) before filtering through a Whatman No. 42 filter. Because soil MBP includes organic P (Po) and inorganic P (Pi), the soil extracts were digested to transform the Po into Pi before measuring the MBP concentrations with the ascorbic acid molybdenum blue method [26]. Specifically, the digestion method of Wu et al. [28] was used. Next, another 10.0 g of the non-fumigated moist soil was extracted with the sodium bicarbonate method before determining the P concentration as described above. Finally, MBP was calculated by subtracting the non-fumigated soil P concentration from the fumigated soil P concentration before dividing by the K value (K = 0.4). In addition, the recovery of the added KH_2_PO_4_ was measured and used to correct the MBP measurements [29].

In addition, MBC and MBN were measured with the chloroform fumigation-extraction method [30], [31]. First, 10 g of the moist soil samples were fumigated for 24 h in ethanol-free chloroform. After chloroform removal, the soil samples were extracted with 40 ml of a K_2_SO_4_ (0.5 M) solution for 30 min by shaking at 300 rpm. Meanwhile, 10 g of the moist soil samples from the non-fumigated soils was extracted directly with 40 ml of a K_2_SO_4_ (0.5 M) solution under the same conditions. The C in the soil extracts was measured with an organic carbon analyzer. Soil MBC was estimated based on the following relationship: biomass C = *E*c/*k*_EC_, where *E*c is the organic C that was extracted from the fumigated soil minus the organic C that was extracted from the non-fumigated soil and *k*_EC_ was 0.45 [32]. The total N concentrations in the extracts were measured by following the procedure of Cabrera and Beare [33]. Total N was determined with an automated nitrate analyzer. Soil MBN was estimated from the following relationship: biomass N = *E*_N_/*k*_EN_, where *E*_N_ was the total N extracted from the fumigated soil minus the total N extracted from the non-fumigated soil and k_EN_ was 0.40 [34].

Soil organic matter was determined by using 0.5 g of the dried sample for K_2_Cr_2_O_7_-H_2_SO_4_ oxidation (the Walkley-Black method) [35]. Total N concentrations were determined from 0.5 g of the dried soil sample with the Kjeldahl digestion-distillation method. Soil pH (soil : water = 1∶ 2.5) was measured with a pH meter (Precision: 0.01). In addition, soil moisture was measured by drying subsamples overnight at 105 °C. Furthermore, soil temperature was determined in the field with a temperature meter (EcoScan Temp 6, 3-Wire RTD PT100 Temperature Meter With Probe EC-TEM6TEM01R).

### Statistical Analysis

All zones (six vegetation zones with three plots in each vegetation zone) were compared within a one-way analysis of variance (ANOVA). Multiple comparisons within the ANOVAs were conducted with a LSD (least significant difference) post hoc test. A quadratic polynomial model was used to model the relationship between the MBP concentration and elevation. In contrast, the relationship between the MBP concentrations and the other factors were fit with linear regression models. A multiple linear regression was used to model the relationship between the MBP concentration and environment factors (i.e. soil P fractionations, soil moisture, soil temperature, pH, SOM and Total N) in SPSS 13.0 for windows. The method “Stepwise” was used for the varible selection in the multiple linear regression. The stepping method criteria: a varible got the entry if its probability of F was less than 0.05, or the varible was removed if its probability of F was more than 0.051. The microbial element ratios (Microbial C:P ratio and microbial N:P ratio) and the reference ratio (mass ratios) [36] were compared with a one-sample T-test. These analyses were completed in SPSS 13.0 for windows. The MBP and soil P fractionation patterns with altitude were plotted and analyzed with the OriginPro 8.0 software.

## Results

### MBP Distribution with Altitude

**Figure 2:**
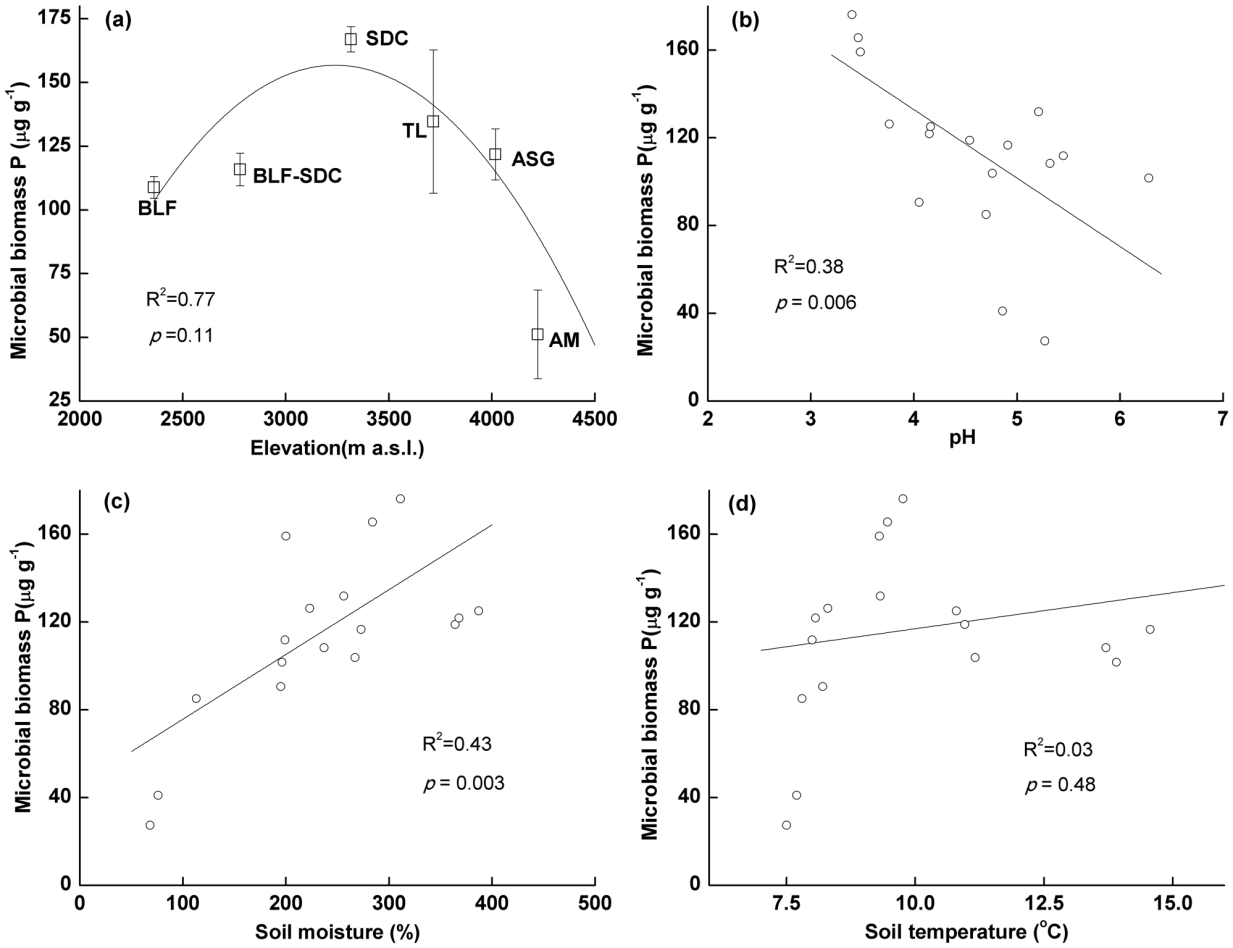
MBP concentrations in the vegetation zones and the relationships between MBP and environmental factors. MBP concentrations were measured in three plots in each vegetation zone (n = 3). The solid line corresponds to the fitted curve of the quadratic polynomial model in Figure (a). A linear regression (n = 18) is shown in Figures (b), (c) and (d).

The distribution of MBP with altitude followed a parabolic trend. The highest MBP (167 µg g^−1^) was observed at approximately 3300 m asl where the subalpine dark coniferous forest is dominant (Fig. 2a). The lowest MBP (51 µg g^−1^) was observed in the AM zone (4221 m asl). In other zones, the MBP varied slightly and decreased between 3300 and 4220 m asl.

### P Concentrations and Fractionation in the Rhizosphere Soils

**Figure 3:**
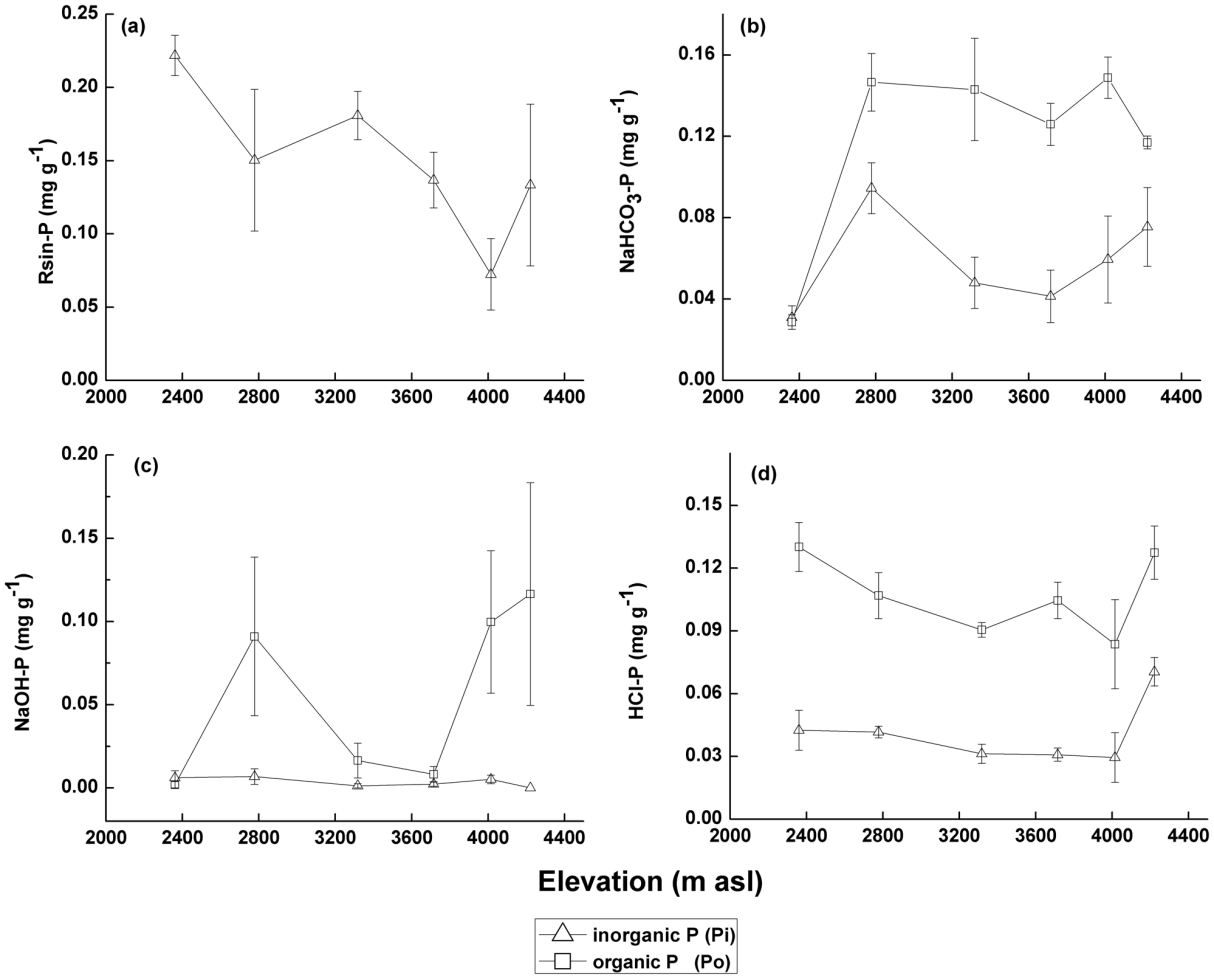
Resin-P(a), NaHCO_3_-P(b), NaOH-P(c) and HCl-P(d) concentrations with altitude. Three plots were sampled in each vegetation zone (n = 3). Vegetation zones along the altitudinal gradient occurred in the following order: BLF, BLF-SDC, SDC, TL, ASG and AM.

P concentrations and fractionation in the rhizosphere soils varied between the different vegetation zones (Fig. 3). For example, the Resin-Pi concentration decreased with increasing elevation in all but the AM zone, in which Resin-Pi slightly increased (4221 m asl) (Fig. 3a).

The NaHCO_3_-Po concentrations were greater than the NaHCO_3_-Pi concentrations in most of the vegetation zones (Fig. 3b). However, these two fractions were similar in the BLF zone (0.029 mg g^−1^ and 0.031 mg g^−1^, respectively). NaHCO_3_-Pi concentrations were greatest at 2800 m asl (BLF-SDC area) and decreased at 3300 and 3700 m asl (SDC and TL zone) before slightly increasing between 4015 and 4221 m asl (ASG and AM zone). The NaHCO_3_-Po concentration varied slightly with altitude.

The NaOH-Pi concentrations were low and consistent relative to the NaOH-Po concentrations with elevation (Fig. 3c). A peak value of NaOH-Po concentration occurred at 2777 m asl (BLF-SDC zone). The low NaOH-Po concentrations occurred at 3317 and 3715 m asl (SDC and TL zone) before increasing at 4015 and 4221 m asl (ASG and AM zone).

The HCl-Pi concentrations in the vegetation zones varied slightly between 2361 and 4015 m asl and increased in the AM zone (4221 m asl) (Fig. 3d). The HCl-Pi, as a single variable, can predict the MBP concentration well (Table 2). The HCl-Po concentrations were greater than the HCl-Pi concentrations in all vegetation zones and decreased between 2361 and 4015 m asl (BLF, BLF-SDC, SDC, TL and ASG zone). In addition, an obvious increase occurred at 4221 m asl (AM zone).

**Table 2:** The results for the multiple linear regression^a^.

						Change	Statistics			
Model	R	R	Adjusted	Std. Error of	R Square	F	df1	df2	Sig F	Durbin-Watson
		Square	R Square	the Estimate	Change	Change			Change	
1	0.757^b^	0.574	0.547	27.763	0.574	21.539	1	16	0.000	
2	0.836^c^	0.699	0.659	24.091	0.125	6.249	1	15	0.025	1.776

a Dependent Variable: MBP; Independent Variable: Resin-Pi, NaHCO_3_-Pi, NaHCO_3_-Po, NaOH-Pi, NaOH-Po, HCl-Pi, HCl-Po, soil moisture, soil temperature, pH, SOM and Total N.

b Predictors: (Constant), HCl-Pi.

c Predictors: (Constant), HCl-Pi, pH.

The results of the multiple linear regression showed that pH and HCl-Pi comprised a good linear model (i.e. less variables, higher R value and R square) which predicts the MBP concentration compared with other variables (Table 2). The model is as follows: MBP = 259-1.32 × HCl-Pi-19.4 × pH, where, MBP is the MBP concentration (µg g^−1^), HCl-Pi is the HCl-Pi concentration (µg g^−1^) and pH is the soil pH.

### Microbial C:P and N:P Ratios in the Different Vegetation Zones

**Figure 4:**
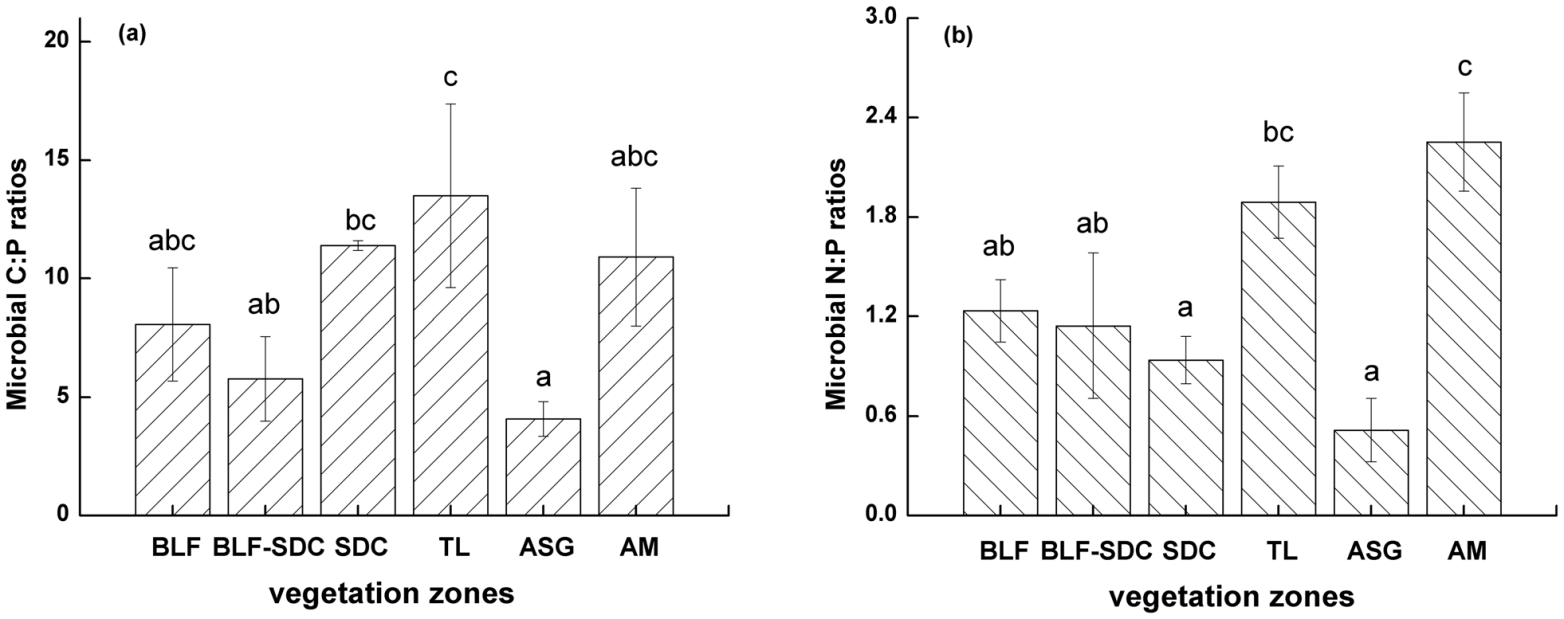
Microbial C:P (a) and N:P (b) ratios in the six vegetation zones. These ratios were calculated on a mass basis (and converted to P based on PO_4_^3−^). The same letters indicate no significant difference (at *p* >0.05). The error bar represents the standard error (n = 3).

In the six vegetation zones, the microbial C:P ratios varied between 5.8 and 13.5 and the microbial N:P ratios varied between 0.5 and 2.3 (Fig. 4). The microbial C:P ratios were the lowest in the ASG zone (Fig. 4) and were significantly different (*p*<0.05) than the C:P ratios in the TL (13.5) and SDC (11.4) zones. The maximum microbial C:P ratio (13.5) occurred in the TL zone, which was significantly different (*p*<0.05) than that in the ASG and BLF-SDC zones. In addition, the microbial C:P ratios in the BLF and AM zones were not significantly different from those in the other zones (*p*>0.05).

The microbial N:P ratio reached its lowest value (0.5) in the ASG zone and was significantly different (*p*<0.05) from the other two adjacent zones (TL and AM zones with 1.9 and 2.3, respectively). The microbial N:P ratio was much greater (2.3) in the AM zone than in the other zones (*p*<0.05).

## Discussion

### MBP Distribution

The MBP concentration may change under different environmental conditions [37]. In the BLF, BLF-SDC, TL and ASG zones, the MBP only varied slightly and it decreased between 3300 and 4220 m asl (from SDC to AM zone) (Fig. 2a). Environmental conditions (e.g., soil type, climate, and vegetation type) are significantly different on the eastern slope of Gongga Mountain. Soil moisture and temperature are two of the most important environmental factors that affect microbial activity [38]. Generally, microbial activity is greater for soil water levels between 50% and 70% of the water-holding capacity [39], [40]. Here, the MBP distribution along the altitudinal gradient followed a parabolic trend with a maximum value of (167 µg g^−1^) at approximately 3300 m asl, where subalpine dark coniferous forests are dominant (Fig. 2a). One possible explanation is that the parabolic distribution in MBP could be related to the distribution of rainfall. The shape of the precipitation curve with increasing elevation was similar to the shape of the MBP curve with increasing elevation. Both peak values were found at approximately 3500 m asl [41]. In addition, our results indicated that a significant relationship exists between MBP and soil moisture (Fig. 2c). Temperature should be positively correlated with microbial activity [42]. However, temperature did not markedly affect MBP distribution in our study (from 2300 to 3500 m asl). One possible explanation for this result is that temperature changes can be buffered by forests (e.g., BLF, BLF-SDC and SDC) [43]. In addition, the high rainfall in these zones results in high soil water contents, which help regulate soil temperature [44]. Furthermore, large glaciers regulate the temperature changes in this region [45]. In addition, although the linear relationship is not significant between MBP and soil temperature taking all the data from all plots into account (Fig 2d), the data from the plots between 3500 and 4200 m asl show the significant linear relationship (R^2^ = 0.69, *p*<0.01, n = 9). This result suggests that temperature becomes a main factor influencing MBP above 3500 m asl.

Because litter is the main nutrition source for microorganisms, P concentrations in plants influence the different MBP concentrations with elevation. At these sites, the P concentrations in the coniferous forests were significantly enriched relative to the P concentrations in the deciduous forests. For example, the mean P concentration in fir twigs was 1258 mg kg^−1^ and the mean P concentration in one type of Rhododendron was only 705 mg kg^−1^ [46]. However, the lowest MBP (51.19 µg g^−1^) occurred in the AM zone (4221 m asl), which was attributed to the cold conditions and sparse vegetation in this zone.

A significant relationship between pH and MBP occurred in the vegetation zones (Fig. 2b), which suggested that low pH values resulted in greater MBP. Some studies have confirmed that microbes can produce acids that decrease pH and releases P from rocks [47], [48].

Rainfall, vegetation type and pH are the main factors that influence MBP concentrations. In addition, above an altitude of 3500 m asl, temperature is an important factor that influences MBP.

### Relationships between MBP and the P Fractions in the Rhizosphere Soil

**Figure 5:**
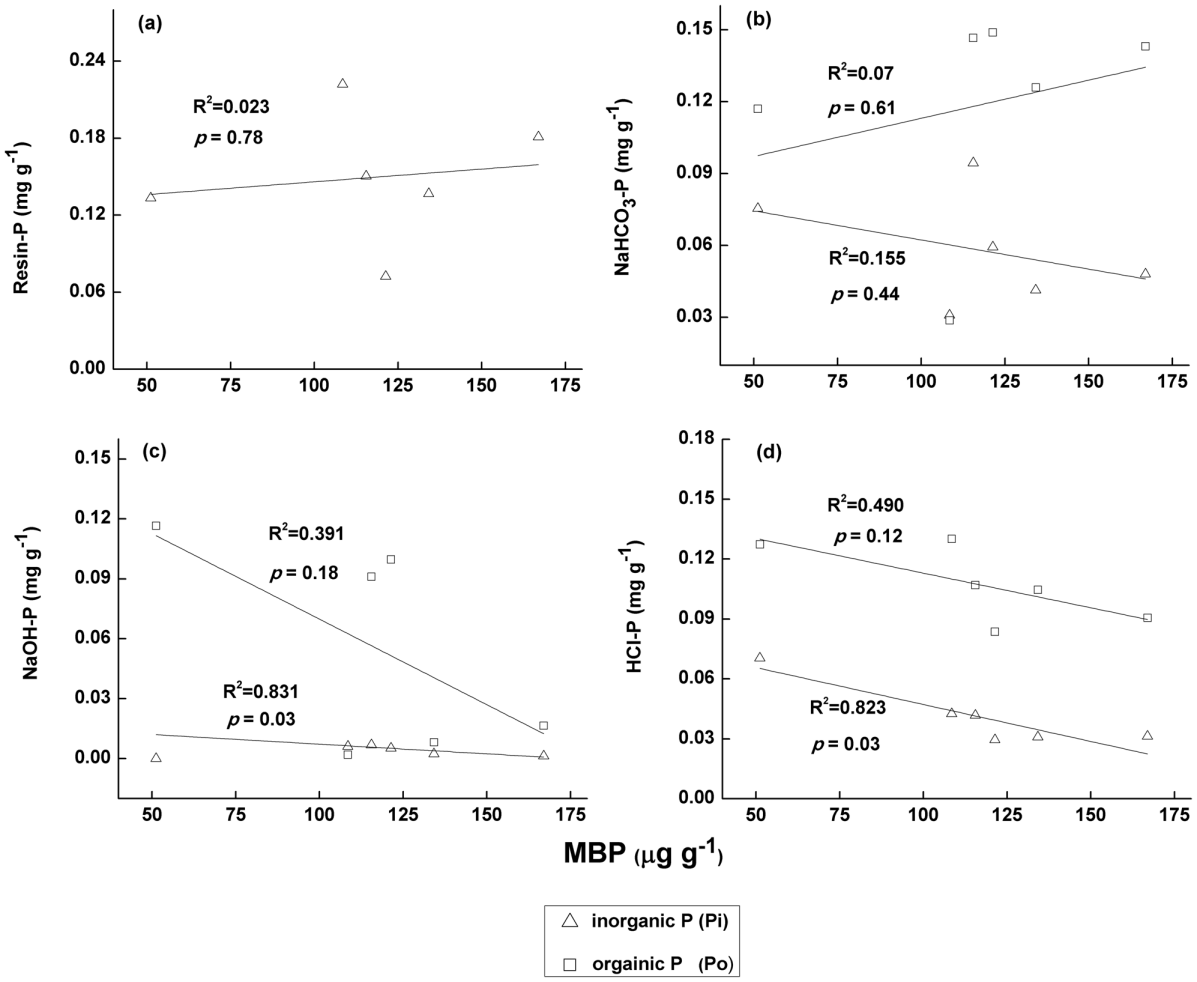
Relationships between MBP and phosphorus speciation on Mount Gongga. (a) MBP vs. Resin-P; (b) MBP vs. NaHCO_3_-P; (c) MBP vs. NaOH-P; and (d) MBP vs. HCl-P. The lines represent linear model fits.

Differences in P bioavailability were determined with the sequential fractionation method based on extractant addition [49]. Generally, the following P fractions were extracted: Resin-Pi, NaHCO_3_-P, NaOH-P and HCl-P [50]. In this study, the Resin-Pi concentrations decreased with elevation in the six vegetation zones (Fig. 3a). In contrast, the Resin-Pi concentration slightly increased in the AM zone (4221 m asl). Resin-Pi is removed from the soil solution with anion exchange resins and is easily absorbed by microbes and plants [51]. However, MBP did not follow this trend (Fig. 3a). The relationship between MBP and Resin-Pi in the rhizosphere soils was not significant (in contrast with results from Chauhan [52] (Fig. 5a). This result suggests that the Resin-Pi concentrations in rhizosphere soils are impacted by multiple factors besides MBP. For example, Resin-Pi can be replenished from other P pools and can be absorbed by roots and microbes [53].

The NaHCO_3_-Po concentrations in the rhizosphere soils of most vegetation zones are greater than the NaHCO_3_-Pi concentrations (Fig. 3b). These results indicated that most of the absorbed P on soil particle surfaces is Po. In addition, the regression analysis demonstrated that the MBP concentrations were positively correlated with NaHCO_3_-Po and negatively correlated with NaHCO_3_-Pi (Fig. 5b). This result suggested that MBP is one of the main sources of NaHCO_3_-Po. In addition, when microbes use absorbed P, NaHCO_3_-Pi is preferred over NaHCO_3_-Po. In addition, some studies have shown that microbial biomass increases after Pi addition [54].

Variations in NaOH-Po concentrations with elevation were more significant than the variations in NaOH-Pi with elevation (Fig. 3c). Thus, NaOH-Po concentrations in the rhizosphere soils were influenced by environmental factors, such as soil type and microbial community structure. In highly weathered soils with high Fe and Al concentrations, their oxides can adsorb (or combine) P to form NaOH-P. Meanwhile, some microbes release more P from AlPO_4_ and FePO_4_ than other microbes [55]. Nevertheless, the degree of weathering in the study area is very low. Most of the soil parent materials were derived from weathered Cenozoic feldspar granite and Permian quartz schist [56]. Therefore, the NaOH-P concentration is potentially controlled by the proportion of phosphate-solubilizing microbes in the microbial community. If the AM zone is excluded (because the NaOH-Pi concentration was below detection limits), a significant linear relationship (p<0.05) occurs between MBP and NaOH-Pi (Fig. 5c). Esberg [57] suggested that the growth of microbes is correlated with NaOH-extractable P, which is an important source of P for microbes. One potential explanation for this significant relationship is that NaOH-Pi is the dominant portion of NaOH-extractable P, which is available to microbial populations across long time-scales.

Although the NaOH-Pi concentration was below the detection limit in the AM zone, the HCl-P (especially HCl-Pi) concentration was much greater than in the other Gongga Mountain zones (Fig. 3d). The linear negative relationship between HCl-Pi and MBP was significant (p<0.05) (Fig. 5d). In addition, the HCl-Pi is an important predictor variable for the MBP concentration in the rhizosphere soil (Table 2). HCl-Pi is primarily extracted from calcium phosphate. In highly weathered soils, Pi predominantly results from Po degradation. In minimally weathered soils, Pi is likely released from mineral phosphate [58]. In the soils in the AM zone, the Al, Ca and Fe concentrations (which can combine with P) [59] were greater than in the other vegetation zones (Table 1). Therefore, mineral phosphate (e.g., calcium apatite) is potentially the main source of P for microbes. In addition, the significant relationship between pH and MBP implies that the phosphate-solubilizing function of microbes likely plays an important role in mineral phosphate use in the AM zone. Furthermore, the temperature and rainfall in the AM zone (4221 m) are relatively low. Thus, the rates of soil weathering and P release are low due to abiotic mechanisms.

A significant linear relationship was identified between MBP and NaOH-Pi (or HCl-Pi) (p<0.05). However, no linear relationship occurred between MBP and Resin-Pi (or NaHCO_3_-Pi). Resin-Pi and NaHCO_3_-Pi are labile and available for plant uptake. However, Resin-Pi and NaHCO_3_-Pi are not supplemented by other P pools in natural ecosystems over relatively short periods. Over long periods, Resin-Pi and NaHCO_3_-Pi may primarily result from microbes (e.g., phosphate-solubilizing microorganisms) and physiochemical weathering processes [49], [51], [60].

Po was the predominant soil P fraction (52.3% of mean total P, Table 1) in the vegetation zones and was more mobile than Pi [61], [62]. Zhou et al. [63] also found that Po were the dominant P fractions at the older sites in Gongga Mountain. These results suggest that Po is potentially an important pool for microbes. In addition, the greater P concentrations (Table 1) and Po ratios suggested that soil weather was relatively weak in the study area [64], [65].

### Microbial C:P and N:P Ratios

Redfield [66] reported a characteristic atomic ratio (mean C:N:P = 106∶16∶1, “Redfield ratio”) in marine water planktonic biomass. Although soil and water environments are different, Cleveland and Liptzin [36] believed that a relatively consistent ratio of elements exists (the “Redfield ratio”) in soil microbial biomass based on their extensive literature review.

The microbial C:P and N:P ratios in this study supported the findings of Cleveland and Liptzin [36], which indicated that the atomic C:N:P ratio in the soil microbial biomass was 60∶7∶1 (atomic ratio) globally and 7.6∶1∶1 as a mass ratio (P conversion based on PO_4_^3−^). Here, the mean mass ratio of C:N:P was 9.0∶1.3∶1.0 (P conversion based on PO_4_^3−^), which was not significantly different from the ratio identified by Cleveland and Liptzin (Table 3). In addition, the altitudinal gradient did not significantly impact the microbial C:P and N:P ratios (Fig. 4a, 4b). The microbial C:P ratio varied between 5.8 and 13.5 and the microbial N:P ratios varied between 0.5 and 2.3 in the six vegetation zones (Fig. 4a, 4b).

**Table 3:** Microbial biomass C, N and P ratios (mass ratios) compared with the reference ratios (mass ratios) proposed by Cleveland and Liptzin.

Ratio	Refer. ratio	Microbial biomass					
		**Min**	**Mean**	**Max**	**n**	**t**	***p***
C:P	7.579	5.766	8.952	13.496	6	0.932	>0.05
N:P	1.032	0.515	1.329	2.525	6	1.592	>0.05
C:N:P	7.579∶1.032∶1		8.952∶1.329∶1				

Refer. ratio is the reference ratio that was proposed by Cleveland and Liptin [36]. Min and Max represent the minimum and the maximum microbial biomass ratios, respectively, in the six vegetation zones. n, t and p refer to sample size, the test statistic and the probability value for the one-sample T test that compared the microbial biomass ratios with the Refer ratios. In addition, the data used in the T test were normally distributed based on the one-sample Kolmogorov-Smirnov test.

The microbial element ratios may vary under different conditions. For example, different vegetation types have different elemental ratios. Microbes may inherit these different ratios by assimilating nutrients from the liter of different vegetation types [67]–[69]. Moreover, our results indicated that the microbial element ratios varied greatly in the vegetation zones above 3500 m asl (TL, ASG and AM zones) and little in the vegetation zones below 3500 m asl (BLF, BLF-SDC and SDC zones) (Fig. 4a, 4b). Vegetation type variations potentially explain this difference. In contrast, climate plays an important role in microbial development and community composition [70], [71]. Thus, the soil microbial elemental ratio can change with the microbial species ratio (e.g., bacterial:fungal ratio) within a community [72], [73].

## Conclusions

The MBP distribution with elevation at Gongga Mountain followed a parabolic trend. pH was an important factor that influenced MBP. Below approximately 3500 m asl, rainfall and vegetation type were the main factors that affected the MBP concentrations. Conversely, above 3500 m asl, temperature, rainfall and vegetation type controlled the MBP distribution at Gongga Mountain.

The Resin-Pi concentrations in the six vegetation zones decreased with increasing altitude. The NaOH-Pi and HCl-Pi concentrations were stable with increasing altitude relative to the NaOH-Po and HCl-Po concentrations. Po was the predominant P fraction in the vegetation zones. However, Pi is likely an important fraction for microbes. Resin-Pi and NaHCO_3_-Pi, which are readily used by organisms, are controlled by many factors.

Generally, NaOH-Pi and HCl-Pi are considered as the non-bioavailable P fractionations. However, compared with the bioavailable P fractions (Resin-Pi and NaHCO_3_-Pi), both NaOH-Pi and HCl-Pi have more significant linear relationships with MBP. The result suggests that the non-bioavailable P fractionations may turn into the bioavailable P with the long-term interaction between microorganism and soil minerals. In addition, HCl-Pi and pH comprised a good linear model which predicts the MBP concentration compared with other variables.

Increasing elevation did not affect the elemental ratios of the microbial biomass (microbial C:P and N:P ratios) in the different vegetation zones. The C:P ratios varied between 5.8 and 13.5 and the N:P ratios varied between 0.5 and 2.3 in the six vegetation zones. The mean C:N:P ratio (the mass ratio) was 9.0∶1.3∶1 (P conversion based on the PO_4_^3−^). Different vegetation types potentially caused these microbial element ratio variations.
